# From detecting abnormalities to diagnosing cancer: how can lay anal examinations be refined?

**DOI:** 10.1016/j.lana.2026.101380

**Published:** 2026-01-29

**Authors:** Ming Wang, Yanru Cao, Qing Zhu

**Affiliations:** Department of Gastrointestinal Surgery, Heze Municipal Hospital, Heze, Shandong, China

We read with interest the valuable study by Nyitray et al.,^1^ which demonstrates that practice can significantly improve concordance between lay anal self-examination or anal companion examination (ASE/ACE) and clinician digital anal rectal examinations (DARE) anal examinations. This work provides important support for the potential of lay examination as a supplementary screening tool.

We would like to offer two considerations for further refining the interpretation and application of these findings, particularly regarding early anal cancer diagnosis.

First, while the study achieved high sensitivity (88%) and specificity (98%) for detecting “any abnormality,” this endpoint included benign conditions such as hemorrhoids. The critical clinical step of differentiating malignant from benign lesions—central to early SCCA diagnosis—was not assessed within the lay examination framework.^2^^,^^3^ Therefore, the impressive detection accuracy for masses may not directly translate to equivalent accuracy in diagnosing cancer.

Second, a deeper analysis of the error cases could greatly enhance future training. Although the false-negative (3%) and false-positive (2%) rates at Visit 2 are commendably low, understanding the specific lesion types involved in these errors (e.g., which were missed or misinterpreted) would be invaluable. Such a lesion-specific breakdown could identify areas for targeted educational reinforcement.

In summary, this study compellingly shows that laypersons can be effectively trained to detect abnormalities. To advance ASE/ACE towards a reliable tool for early cancer detection, future research could beneficially focus on its diagnostic specificity for malignancy and a detailed analysis of error patterns.^4^

## Contributors

M.W.: Conceptualization, Writing—Original Draft, Writing—Review and Editing. Y.C.: Writing—Review and Editing. Q.Z.: Writing—Review and Editing.

## Declaration of interests

We declare no competing interests.
